# Beyond Conventional Treatments: Exploring CAR-T Cell Therapy for Cancer Stem Cell Eradication

**DOI:** 10.1007/s12015-024-10786-4

**Published:** 2024-09-23

**Authors:** Lobna E. Rabie, Ahmed A. Mohran, Kholoud A. Gaber, Nour M. Ali, Asmaa M. Abd El Naby, Eman A. Ghoniem, Basmala A. Abd Elmaksod, Ahmed N. Abdallah

**Affiliations:** 1 Faculty of Pharmacy, Fayoum University, Fayoum, Egypt; 2 Zoology Department, Faculty of Science, Alexandria University, Alexandria, Egypt; 3 Molecular Biology and Chemistry Department, Faculty of Science, Alexandria University, Alexandria, Egypt; 4 Chemistry Department, Faculty of Science, KFS University, Kafr El-Sheikh, Egypt; 5 Zoology-Chemistry Department, Faculty of Science, Beni Suef University, Beni Suef, Egypt; 6 Biotechnology and Chemistry Department, Faculty of Science, Alexandria University, Alexandria, Egypt; 7 Zoology-Chemistry Department, Faculty of Science, Mansoura University, Mansoura, Egypt; 8 Hormones Department, Medical Research and Clinical Studies Institute, National research Centre, Cairo, Egypt

**Keywords:** Immunotherapy, Cancer stem cells, Chimeric antigen receptor T cells, CSC antigens

## Abstract

**Background:**

For decades cancer remained the center of attention in the scientific community as its survival rates are low. Researchers from all around the world wanted to know the core of the problem as to what initiates cancer in a patient and helps with its progression. Many postulations came to light, but Cancer Stem Cells (CSC) was the most appealing and convincing.

**Main Body:**

In this review, we shed light on a potential solution to the problem by reviewing CAR-T cells (Chimeric antigen receptor T cells). These specialized T cells are designed to detect specific antigens on cancer cells. We analyse the steps of their formation from the collection of T cells from the patient’s bloodstream and modifying it to exhibit specific CAR structures on their surfaces, to reinjecting them back and evaluating their efficacy. We thoroughly investigate the structure of the CAR design with improvements across different generations. The focus extends to the unique properties of CSCs as in how targeting specific markers on them can enhance the precision of cancer therapy.

**Conclusion:**

Despite the successes, the review discusses the existing limitations and toxicities associated with CAR-derived therapies, highlighting the ongoing need for research and refinement. Looking ahead, we explore proposed strategies aimed at optimizing CAR-T cell therapy to mitigate adverse effects for improved cancer treatments.

**Graphical Abstract:**

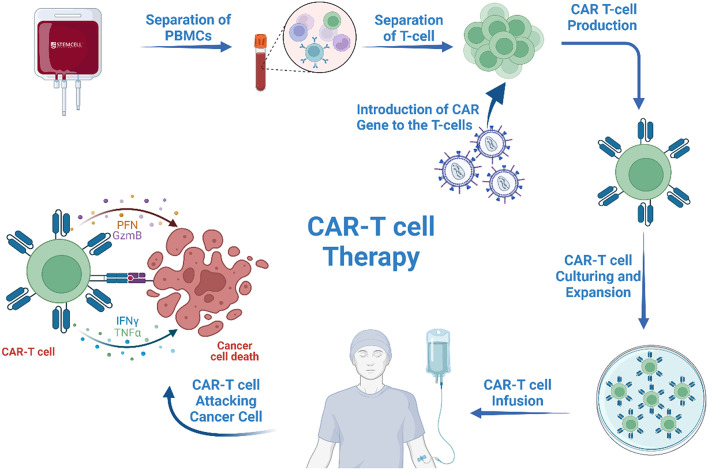

## Introduction

About 150 years ago, it was hypothesized that cancer develops from “stem cells” and this hypothesis was supported by a growing body of experimental data that changed perceptions of tumorigenesis and cancer cell biology [1]. A distinctive subgroup of cancer cells was discovered, called the cancer stem cells (CSCs), which can self-renew and specialize in many cell types. Multilineage differentiation is essential for the development and progression of cancer which induces neoplastic progression, metastasis, recurrence, chemoresistance, radiation resistance, and other malignant phenotypic traits [1]. CSCs exhibit several hallmarks including causing new tumor formation with a small number of CSCs [2] and increased activation of DNA repair mechanisms as a result of the expression of certain factors or surface markers [3]. It was known that Immune cells are constantly searching for and destroying cells that have turned into cancer. However, some altered cells can evade immunity and eventually develop tumors. This means that CSCs may also exhibit immune camouflage as a malignant trait. It was noted that normal stem cells possess immune-modulatory qualities [1] and are capable of evading the immune system via several mechanisms. Only a few CSC-targeted strategies have been applied to cancer therapy to date. These involve making CSCs more sensitive to conventional medications, incentivizing them for differentiation, eliminating their niches, and also targeting and blocking critical CSCs’ signaling pathway elements [4]. However, there are other more specific ways to target CSCs, and one of these is immunotherapy. Immunotherapy is mainly used to increase immune function through immune microenvironment regulation, which allows the immune system’s cells to target cancer cells and kill them [5].

Since CSCs can escape from the immune system, modifications have been made to eliminate CSCs. This is done by adding a unique receptor to T cells known as “chimeric antigen receptor” which targets a specific marker on the surface of CSCs, so it is considered a type of “cell-based gene therapy”. This type of receptor enables T-cells to recognize and terminate CSCs specifically [5]. In CAR T-cell treatment, T-cells extracted from the bloodstream of a patient are remodeled in the laboratory by adding specific genes that are translated to the CAR which in turn facilitates the binding to a certain antigen on tumor cells. Each CAR is developed for a particular malignancy since various cancers have various antigens [2, 5].

In summary, this review aims to go over how CAR T-cell therapy deals with CSC resistance to eradication by passing through the possible markers that could be targeted on their surface along with the main CAR T cell products authorized by the FDA. Nevertheless, CAR-derived therapies exhibited astounding success, especially in blood malignancies. This review discusses the main adverse effects and constraints of this therapy as well as providing several approaches to enhance their efficacy.

## Principles of Formation

### Production

In order to get an efficient CAR-T cell product, a sequence of many steps need to be carried out adequately. First, we need to obtain the T cells themselves, we do this by the leukapheresis technique [6] followed by centrifugation of the product using density-gradient centrifugation method to get peripheral blood mononuclear cells (PBMCs) [7]. Then to isolate the T cells we need to either apply Magnetic-Activated Cell Sorting (MACS) [8] or Fluorescence-Activated Cell Sorting (FACS) [9] in order to target the T cell population that we desire. And since every T cell population has different functions, the choice of which population to use for the personalised therapy doesn’t only depend on the characteristics of the specific T cell population, but also the patient’s unique requirements and medical history [10]. Additionally, the origin of the T cell itself can vary, being either taken from the patient directly (autologous product), or from a third-party donor (allogenic product) [6].

Right after obtaining the accurate T cell population from its origin, we activate the T cells. T cell activation plays a critical role in the efficiency of manufacturing because it has a direct influence on the Transgene introduction [6]. The activation process is achieved by more than one method that work on the stimulation of CD3 and CD28, and the most popular method is using paramagnetic beads coated with anti-CD3 and anti-CD28 imitating a cancer cell with these surface antigens [11]. The CAR transgene is then introduced into the T cells, and the most used means is the viral transduction through lentiviral or retroviral vectors [6]. There is a wide array of differences, but most notably that lentiviral vectors can transduce the transgene into nondividing cells, on the other hand γ-retroviruses only work on dividing ones [6]. Also, the region where they imbed their construct differs as lentiviruses on a bigger scale work on introns and intergenic regions, while γ-retroviruses insert in promotors, exons, and untranslated regions UTRs [10]. Alternatively, the usage of nonviral methods have also been explored. The most common means are CRISPR-Cas9 [12], transposons, especially the Sleeping Beauty transposon [13], and RNA-based transfection [14].

Finally, after the introduction of the CAR transgene into the T cell, we need to expand and proliferate the number of the cells to meet the criteria of the administered dose [10]. Cytokines play a critical role in the expansion of T cells such as IL-7 and IL-2 [10, 15]. And while the main purpose for the expansion process is to increase the number of the administered cells, it also plays a critical role in the depletion of other non-T cells present in the media [10]. The proliferation step takes approximately 1 to 2 weeks and afterwards we would have obtained the final T cell product [10]. However, prior to administration the product must undergo quality protocols to ensure multiple standards that were set by the FDA. These include safety, purity, potency, and stability of the product [16, 17].

**Fig. 1:**
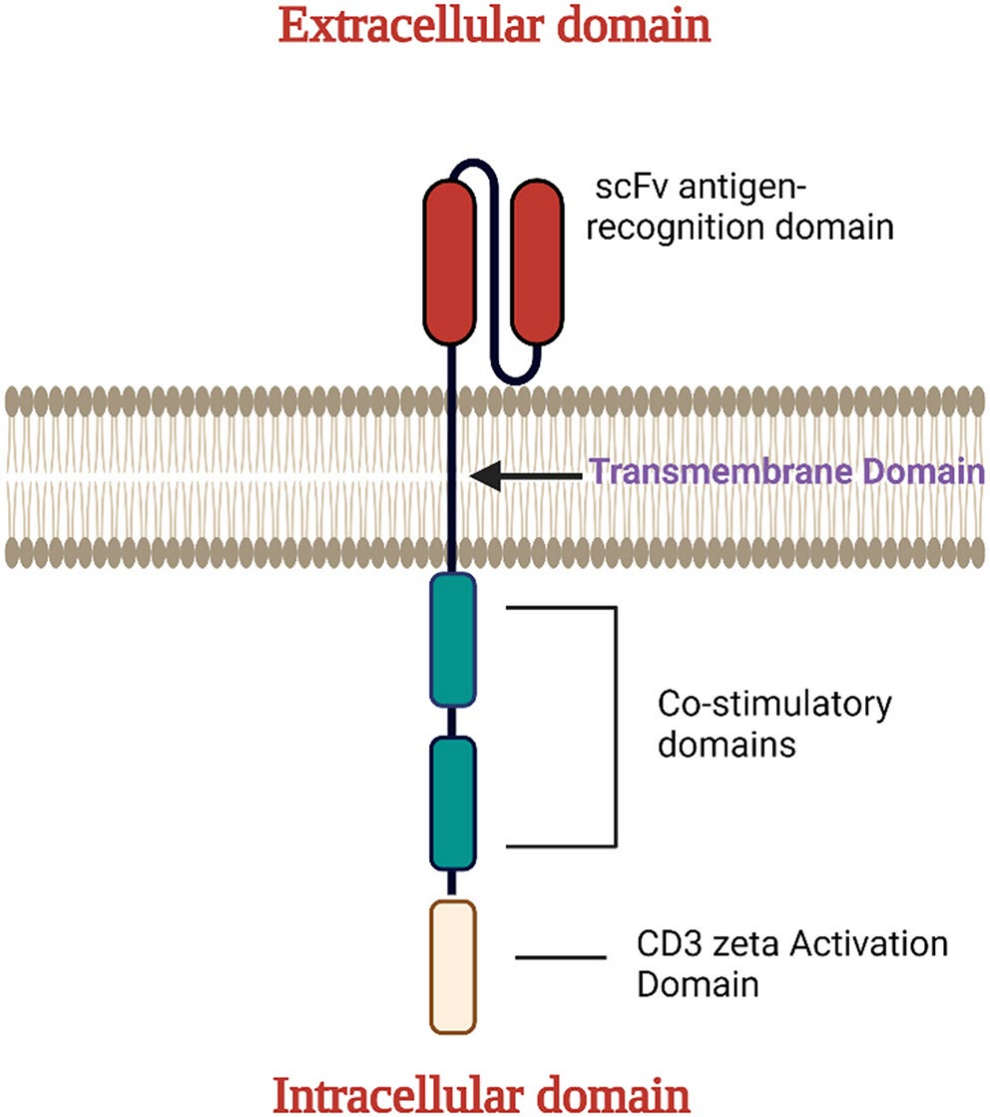
The primary components of the CAR structure are shown in this figure. These elements include the extracellular domain, which recognizes antigens, the intracellular domain which contains CD-3 signaling domain and one or more costimulatory molecules, and finally, the transmembrane domain which connects the two previous parts

Three main components constitute the CAR structure, those are the extracellular and intracellular domains, along with the transmembrane domain [11]. Firstly, the extracellular and intracellular domains make up the initial portion of CAR composition; its main function is to spot a certain antigen and is primarily extracted from a (scFv) which is the antigen recognizing and binding portion of the CAR construct, which is produced by utilizing the monoclonal antibody’s lightweight and bulky chains and connecting them using a component called “the linker” (Fig. 1).

Secondly, the transmembrane domain which is propagated within the membrane of the cell and keeps CAR’s extracellular and intracellular domains united. It is well known that this part is generated from CD8 or CD28 and functions as a pivot for transmitting signals to the intracellular domain.

Finally, the intracellular domain which is obligatory for T-cell activation and constructed of CD3 and one or more co-stimulatory portions [18]. It was common knowledge that Co-stimulatory molecules are mandatory for the enlargement and functioning of the CAR-T cells. 4-1BB and CD28 are considered the two main costimulatory domains utilized in the construction of CARs. In several trials, it was established that CARs including the CD28 or 4-1BB intracellular domain exhibited a comparable proportion of responses among patients with malignancies of the bloodstream. It was noted that these different responses are due to the variation in T cell persistence between the two types of CARs. These T cell resistance variables initially became apparent in clinical investigations employing competing examinations on CAR-T cells owing to the two main co-stimulatory domains CD28 and 4-1BB in animal models. Results on B cell tumors confirm that CD28-derived CAR-T cells become observable throughout few weeks, but 4-1BB-derived CAR-T cells may survive inside individuals for a long while without getting medical care. Finally, in clinical research, CD19-targeted CAR-T cells carrying the 4-1BB stimulatory domain indicate more durable persistence than those with CD28. It was hypothesized that 4-1BB persistence is connected to increased antiapoptotic substances and decreased T-cell depletion, especially in comparison with CAR-T cells with CD28.

### CAR Generations

CARs are bioengineered receptors that are highly selective against a particular antigen. Three decades ago, the first CARs were created and since then, their development has progressed gradually. they can recognize antigens even without depending on the major histocompatibility complex (MHC) because MHC is down-expressed in many types of cancers [6].

**Fig. 2:**
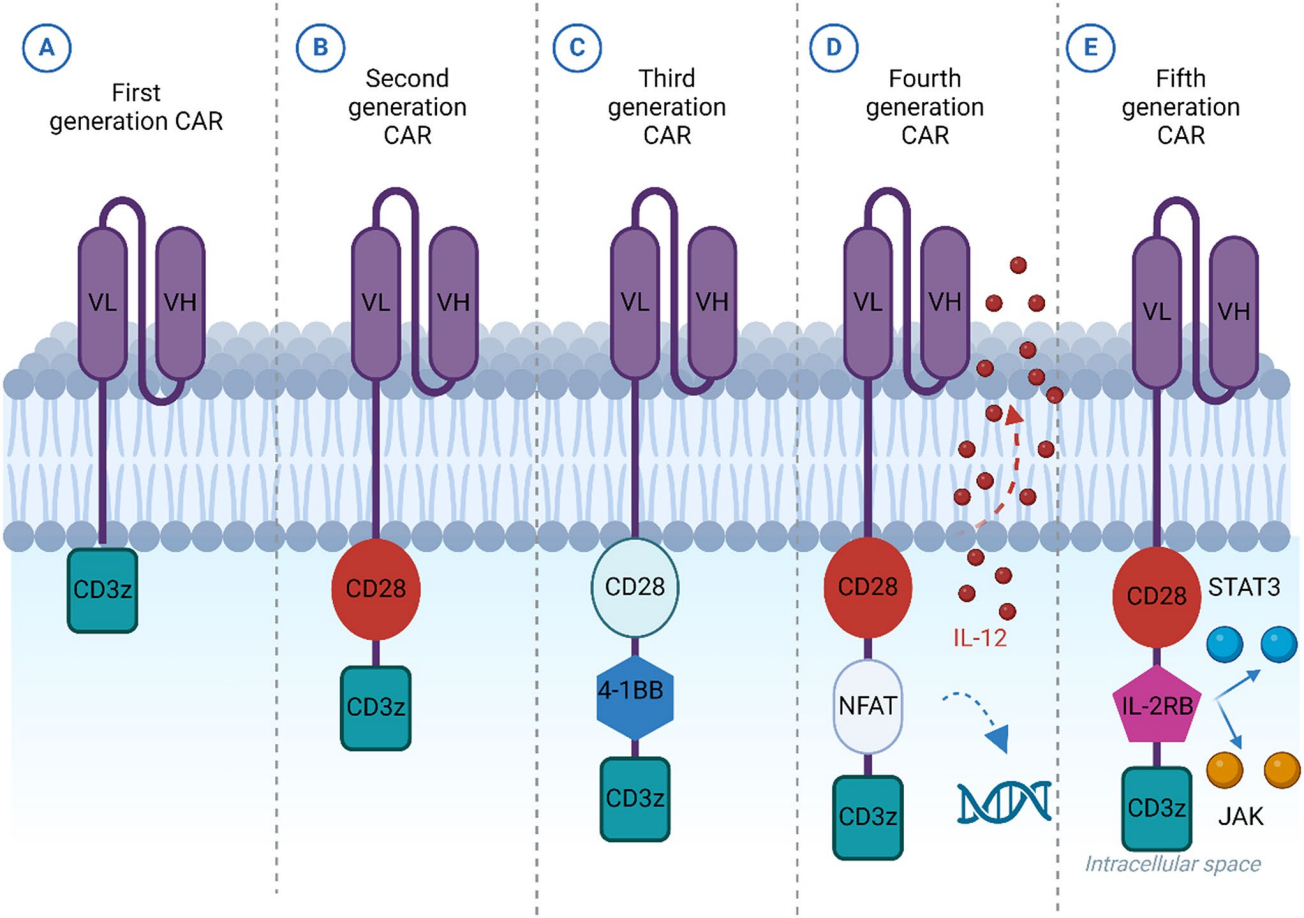
This figure shows the five CAR generations: (**A**) demonstrates that the only component of the first generation of CARs is the CD3ζ signaling domain, which is used alongside the extracellular scFv to stimulate T cells. (**B**) The CARs of the second generation were designed using an extra costimulatory domain. This domain involved CD28 or 4-1BB which enhance the cytotoxicity, proliferation, and long-term response of CAR-T cells. (**C**) To strengnthen T cell persistence, two more costimulatory domains, such as CD28, 41BB, ICOS, and OX-40, are added to third-generation CARs. (**D**) By releasing IL-12, fourth generation CARs have the ability to induce other T cells to produce granzyme B, perforin, and interferons; moreover, it has the capacity to prevent the T-regulatory cell division. (**E**) The fifth CAR generation contains CD34, CD28, IL2RB, and STAT3 inducer to improve CART activation, proliferation, and survival

Based on the construction of the Intracellular signaling domains, the evolution of CAR design during the previous 30 years can be classified into 5 generations (Fig. 2) [19]. The first generation solely consists of the signaling endodomain CD3ζ, which is combined with extracellular scFv to induce T-cell modification and activation [20]. To give the T-cell additional signals, CARs of the second generation appended an intracellular signaling domain that consists of CD3ζ and several co-stimulatory proteins. It includes 4-1BB or CD28, which boost the cytotoxicity, proliferation, and long-term response and increase the CAR-T cells’ in vivo lifespan [11]. Two additional costimulatory domains such as OX-40, CD27- CD28, ICOS, and 41BB are added to the third generation of CARs to enhance the cytocidal potential parallel to the longevity of T-cells [21]. The fourth generation’s idea was initiated to defeat the immunosuppressive tumor microenvironment (TME) for individual patients. Provided that, it is genetically engineered to defeat the generated immunosuppressive TME by releasing IL-12 which has the ability to induce T cells to produce IFN, perforin, and granzyme B; added to that, it has the capacity to prevent the proliferation of Treg. Finally, the fifth CAR generation contains CD34, CD28, IL2RB, and STAT3 inducer to improve CART activation, proliferation, and survival [22].

## Candidate Cancer Stem Cell Antigens Recognized by CAR-T Cells

### CD133

CSCs in brain, breast, liver, and colon cancers mainly have the membrane-bound glycoprotein CD133. CD133 is responsible for cancer resistance to chemotherapy and cancer recurrence. It has been shown that the increased expression of CD133 in glioblastoma is correlated with the activation of “protein kinase B” and “B-cell lymphoma-2” pathways which are responsible for CSC survival [23]. The infusion of CD133 CAR T-cells with cisplatin was attested that it markedly decreased the weight and volume of gastric cancer xenograft tumor models and eliminated CD133 positive CSCs-like cells. In the same study, it was observed that cisplatin is able to raise the population of CD133-positive cancer stem-like cells which are then targeted by CD133 CAR-T cells, so this combination could improve gastric cancer prognosis (Table 1) [24].

**Table 1:** CSCS antigens: this table includes the main CSC marker targeted by CAR-T cell therapy in several clinical trials with their main findings

CSC marker	CAR generation	Cancer	Main findings	References
CD133	NA	Hepatocellular carcinoma	Of 21 patients: One patient, partial response. 14 patients, stable disease. Seven patients, progressive disease. Median OS: 12 months Median PFS: 6.8 months	NCT02541370 [57]
MUC-1	Third and Fourth generation CARs	MUC-1 positive metastatic seminal vesicle cancer	Patient number: one patient This trial reported: A 10-fold increase in IL-6 60% increase in TNF-α SM3-CAR T cells exhibited slight tumor necrosis. PSM3-CAR T cells exhibited marked tumor necrosis.	[58]
EpCAM	Second generation CARs	EpCAM positive cancers	No results posted	NCT03013712
c-Met		Melanoma and breast cancer	Patient number: four breast cancer patients and three melanoma patients. Grade 1–2 toxicities: six patients. Grade 1 CRS: one patient. No grade 3 or more toxicities. Stable disease: four patients. Progressive disease: three patients.	NCT03060356 [59]
CD-20/CD-19	Second generation CAR	Relapsed B-cell malignancies	Patient number:22 patients Dose: 2.5 × 10^6^ cells per kg One patient: grade 3–4 CRS. Three patients: grade 3–4 neurotoxicity. ORR (12 patients): 100% CR: 92% PR: 8%	NCT03019055 [60]
CLL-1	NA	Relapsed/refractory acute myeloid leukemia	Patient number: 10 patients Seven patients developed CRi (70%). 10 patients emerged CRS and severe pancytopenia. Two patients died of severe infection.	[61]
LGR-5		Colorectal cancer	No results posted	NCT05759728

### EpCAM

Epithelial cell adhesion molecule is an integral membrane glycoprotein and is appraised as a prevailing marker for CSCs in numerous types of cancer-like prostate, breast, and hepatocellular carcinoma (HCC). It’s clear that EpCAM facilitates the proliferation and metastasis of numerous types of cancers [23, 25] It was observed that EpCAM-CAR T-cells can eradicate EpCAM^+^ cancer cell lines and release remarkable levels of interferon- α (INF-α) and interferon- γ (INF-γ) in vitro and inhibited cancer growth in colon animal models in vivo [25]. It was shown in a different study that EpCAM-CAR T cells have significant killing effects on PC3M prostate cancer cell line and significant inhibition of cancer growth in vivo [26].

### CD44

CD-44 is a surface glycoprotein that participates in cell migration and metastasis. Both solid and hematological tumors predominantly express CD44 variation 6. Studies revealed that CD44v6 CAR T cells possess higher persistence and expansion than CD19 CAR T-cells and have the capacity to slow tumor progression in ovarian and lung carcinoma tumor models [27]. Another study developed a CD-44 CAR-T cell using a non-viral vector which improved the expression efficacy and reduced the viral toxicity. In addition to that, CD-44-CAR-T cells exhibited diminished cancer mass and increased survival in HCC xenograft mice [28].

### 3.4. CLL-1

Human c-type lectin-like molecule (CLL-1) is a type-2 integral membrane glycoprotein that is linked with tumor recurrence and chemoresistance. The presence of CLL-1 is limited to myelogenic lineage and AML blasts especially on Leukemic-Stem Cells (LSCs) with low expression on Hematopoietic Stem Cells (HSCs), so it is considered an ideal marker for AML [23, 29]. CLL-1 CAR T cells are markedly recognized for inhibiting cancer progression in AML tumor models in mice without targeting normal HSCs due to the absence of CLL-1 expression on them [29]. In another clinical trial conducted on four children with refractory/relapsed AML, three patients revealed complete remission (CR) and minimal residual disease negativity (MRD), but one patient developed GVHD making it obligatory to conduct further studies to approve the safety of CLL-1-CAR T-cell therapy [30].

### TIM-3

T-cell immune-globulin mucin-3 (TIM-3) is an outer membrane glycoprotein type 1 which is mainly expressed in AML blasts and LSCs. TIM-3 is considered a coinhibitory receptor for regulating the activity of INF-γ secreting CD4^+^ and CD8^+^ cells. It also has been shown that the TIM-3 receptor activates LSCs self-renewal upon binding with its ligand galactin-9 due to the stimulation of nuclear factor kappa B (NF-kB) and β catenin signaling pathways [23, 31]. Anti-TIM-3 CAR T cells proved their efficiency in eradicating LSCs and reducing tumor size in AML xenograft tumor models. Also, the same study reported that the presence of TIM-3 on regulatory T-cells and M2 macrophages has the advantage of diminishing their immunosuppressive effect on cancer [31]. In another study, they created a bispecific CAR T cell recognizing both TIM-3 and CD13 to avoid the off-tumor toxicity induced by targeting CD-13 alone which is expressed in HSCs and other normal cells [32].

### Siglec-6

Sialic acid-binding immunoglobulin-like lectin-6 is a type of surface protein family that is involved in the inhibitory signaling of immune cells. Siglec-6 has a unique feature different from other siglec family members such as being predominantly expressed in AML blasts and absent in HSPCs. It was recognized that siglec-6 CAR T-cells were efficient in decreasing leukemia burden and prolonging survival in AML xenograft models with no significant toxicity on HSCs [33].

## FDA Approval of CAR-T Cell Therapies for B-Cell Lymphoma

**Fig. 3:**
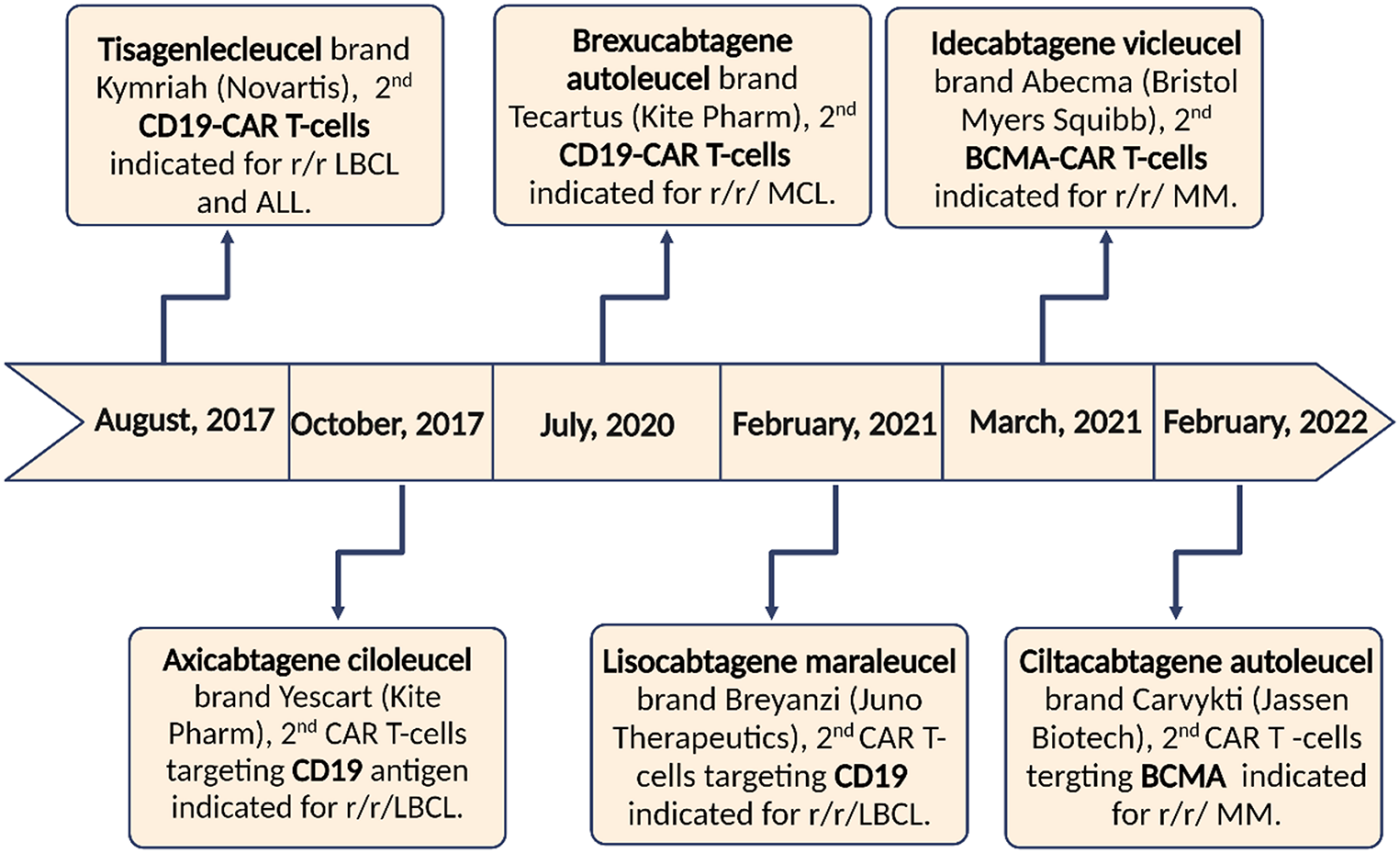
FDA-authorized CAR T cell therapies. This figure highlighted the main CAR T-cell therapies that approved over the 2017 to 2022 period providing details including an example for each product, the manufacturer, and the indication for each treatment. r/r LBCL (Refractory and Relapsed Large B-cell Lymphoma), BCMA (B-cell Maturation Antigen), MM (Multiple Myeloma) MCL (Mantle Cell Lymphoma)

Seven years ago, the FDA authorized several CAR T-cell products to treat blood cancers (Fig. 3). CD19 is thought to be the first antigen targeted by CAR T-cell therapy to cure relapsed/refractory nonhodgkin lymphoma (r/r NHL) with large B-cell lymphoma (LBCL) as their predominant type. The first product, Axicabtagen (Axi-cel) is a CD19 CAR T-cell with CD28 as a co-stimulatory molecule. Axi-cel was tested to treat 101 patients with r/r-LBCL after using two or more treatment regimens and it was approved in 2017 after achieving an object response rate (ORR) of 82%. However, 93% of the patients who received axi-cel showed signs of cytokine release syndrome (CRS) making it better suited for young patients who don’t have other comorbidities [34–36].

The second one, Tisagenlecleucel (Tisa-cel) is also a CD19 CAR-T cell but with a 4-1BB as a costimulatory molecule. 111 LBCL patients were enrolled to be treated with tisa-cel and they showed an ORR of 52%, but only 58% of patients suffered from CRS. Based on these results, tisa-cel was approved in 2018 by the FDA and EMA for adult patients enduring LBCL. It was observed that the lower risk of CAR T-cell adverse effects of tisa-cel is attributed to the presence of the 4-1BB costimulatory molecule [35–37].

The last CD19-CAR-T cell product is Lisocabtagene (Liso-cel) still including a 4-1BB co-stimulatory molecule similar to tisa-cel but with a different method of manufacturing. Regarding liso-cel, 269 patients were selected according to criteria that are different from those used in previous trials so that, it covered LBCL patients with other comorbidities such as kidney dysfunction and heart failure. This study recorded promising results with ORR of 73% and CRS noted only on 42% of the patients. Arising from these data, liso-cel was authorized in 2021 by the FDA for both adult and young patients with LBCL [35, 36].

In addition to CD19, there is another antigen called B cell maturation antigen (BCMA) that is utilized in creating two CAR T-cell products called idecabtagene and ciltacabtagene. In 2021 and 2022, respectively, the FDA licensed both of these products to treat r/r multiple myeloma [35].

## Impeding Toxicities and Limitations

### Antigen Loss

CAR-T cells are manufactured to target a certain antigen on the tumor surface, in some cases the targeted malignant cell can partially or completely lose the surface antigen allowing the cancer cell to remain undetected from the T-cells. This phenomenon is known as “Antigen Escape” or “Antigen Loss” [38, 39]. Recent studies revealed that CAR-T cell therapy is efficient with cases which refract/ relapse from other modes of treatment. Nevertheless, around half of them show recurrences of the tumor [38]. This can happen with antigens like CD19, BCMA, IL13Ra2 [38]. The common feature between the CAR-T cells that induce relapse is that they all have 4-1BB as the costimulatory domain [40]. On the other hand, the tumor cells may undergo mutations or splicing in the protein genes, or they may switch lineages, all of which result in the formation of chimeric proteins that closely resemble the primary targeted proteins, but unidentical that the CARs cannot recognize them [41].

“Epitope spreading” is a technique used to overcome this problem as it results in targeting more than just one antibody [39]. The usage of “multispecific CAR-T cells” has proven useful in detecting more than one antigen, so if one escapes, another one will be targeted [39]. A recent study suggests that targeting a toxin expressed by the cancer cells rather than an antigen would result in no antigen loss due to the stability of the toxin structure [42]. A different study suggests the usage of CAR-T cells in conjunction with another factor that promotes Fas expression on cancer cells. This way we can eliminate antigen escape and tumor cell heterogenicity [40].

### On Target off Tumor

When antigens on tumor tissue are also expressed on healthy tissue, it gives the CAR constructs a chance to target and eliminate normal cells. This toxicity is called “on-target off-tumor” [38]. It came to light after the passing of a patient with lung cancer, whose treatment by CAR-T cells made it worse as the T cells attacked healthy lung epithelia expressing the same tumor-targeted antigen [43]. The primary reason for its occurrence is the low antigen specificity in solid tumors; meaning that rarely can we find an antigen that’s only expressed in tumor cells and not normal ones [44].

Methods to overcome this toxicity involve dual/multispecific CAR-T cells that, in order to work, have to detect two or more antigens on the tumor cell [45]. Another addition to this method is using a NOT gate that includes an inhibitory CAR and a killing CAR and on the same T cell [46, 47], and this method is discussed in detail further in the review. Normally, tumor cells show high affinity to their antigens, so by using lower affinity scFv fragments on the CARs, we can help direct the T cells toward tumor cells rather than normal cells that normally show low affinity [40]. Moreover, the usage of a suicide gene system encoded within the CAR-T cells’ genes can prove effective in eliminating the out-of-control T cell upon contact with a normal cell [43].

### CAR T-Cell Trafficking, Tumor Infiltration, and Tumor Microenvironment

**Fig. 4:**
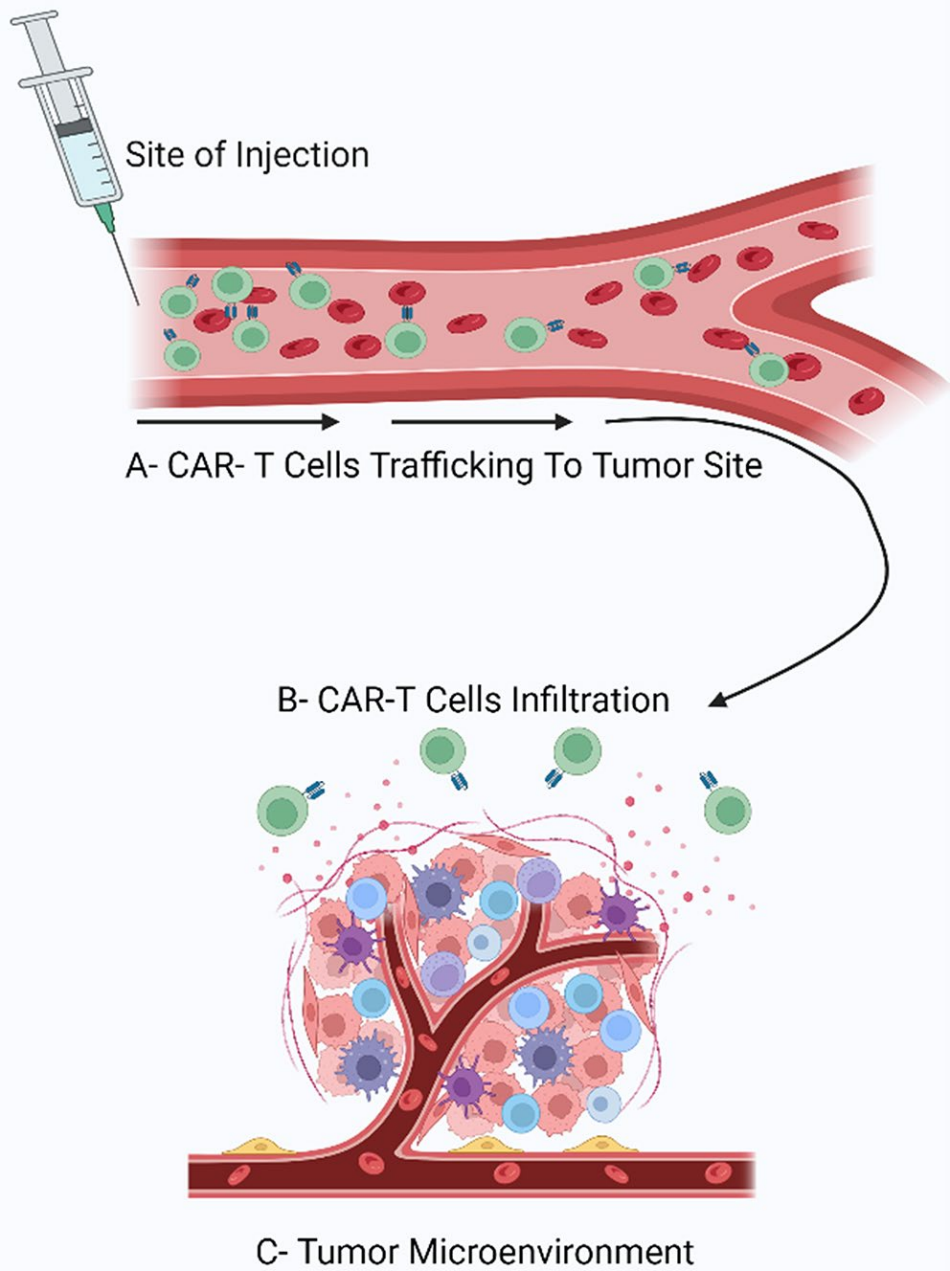
This figure summarizes the steps that CAR-T cells undergo from the on-spot of injection till reaching the tumor site. (**A**) Trafficking: After injection, the CAR-T cells start circulating in the patient’s bloodstream till reaching the tumor site. (**B**) Infiltration: After CAR-T cells arrival they have to enter into the tumor cells and only in solid tumors do they have to overcome (**C**) the TME that promotes the growth of the tumor by supplying it with nutrients through special blood vessels, it also secretes cytokines that repel immune cells away, this gives the TME it’s immunosuppressive property

After injection with CAR-T cells, they have to end up at the tumor site so that they can function (trafficking) [38, 45]. Contrary to hematological malignancies, and as discussed in Fig. (4), in solid tumors they face what is called the TME “tumor microenvironment” which consists of all the surrounding blood vessels, ECM, and immune cells around the tumor [38, 45, 46]. When it comes to tumors, TME is immunosuppressive as it contains cytokines that can inhibit the CAR-T cells’ activity [45]. For the T-cells to prove effective, they have to enter this microenvironment and contact the tumor cells (Infiltration) [40].

Routes of delivery other than systemic delivery are being investigated to ameliorate the trafficking effects. For example, local administration of the T-cells can notably decrease their chances of swaying away from the target tumor site [38]. Another possible route would be the intra-tumoral route that showed significant success in a study conducted on Xenograft mice [48].

Solid tumors naturally express chemokines; however, owing to the absence of the corresponding chemokine receptors on the surface of CAR-T cells, it’s strenuous to traffic and infiltrate inside of the tumor [38]. To counter that, we use the T-cell Redirected for Universal Cytokine Killing (TRUCK) designs that not only are they designed to target and kill cancer cells but also produce specific cytokines to improve the immune response against tumors [46]. One study evaluated the efficiency of the CAR-T cells after expressing a CXCR2 receptor on their surface that matches the CXCL1 chemokine, and they found that it enhanced their trafficking and infiltration [48, 49].

### Cytokine Release Syndrome

When the required immune response is activated upon CAR-T cell administration, cytokines are released. Now when these cytokines exceed the normal expression level, they cause what is called CRS (Cytokine Release Syndrome) that resulting in overactivation of the immune response causing systemic inflammatory responses [43]. This includes almost all the systems of the body and may cause respiratory failure, renal failure, hepatic dysfunction, cardiac dysfunction, neurotoxicity, and other minor symptoms such as fever, skin rash, vomiting, and nausea [41, 43]. It may be life-threatening if not treated timely. In mild cases, antibiotics and antipyretics are used, while in severe cases tocilizumab would be the best option as it counters IL-6 [42] ameliorating the symptoms in a matter of hours without compromising the efficiency of CAR-T cells [40, 43].

### Tumor Lysis Syndrome TLS

When the CAR-T cells target and eliminate numerous tumor cells within a short time span, we shouldn’t be happy just yet because this causes tumor lysis syndrome (TLS). This syndrome happens as a result of the excessive release of the cellular content of tumor cells into the bloodstream [43]. This leads to a sudden increase in levels of uric acid, potassium, and phosphorus. And if left untreated it will result in heart failure, kidney failure, or even death in severe cases [43].

Blood tests [43] should be considered along with assessing the tumor burden [50] prior to administration of CAR-T cells. Moreover, preventative measures should be taken like hydration and using of agents that decrease the uric acid in the blood like rasburicase, febuxostat, and allopurinol [50].

## Current Advancements and Future Prospectives of CAR-T Cell Therapy

### Minimizing CAR-T Cell-Induced Toxicity

**Fig. 5:**
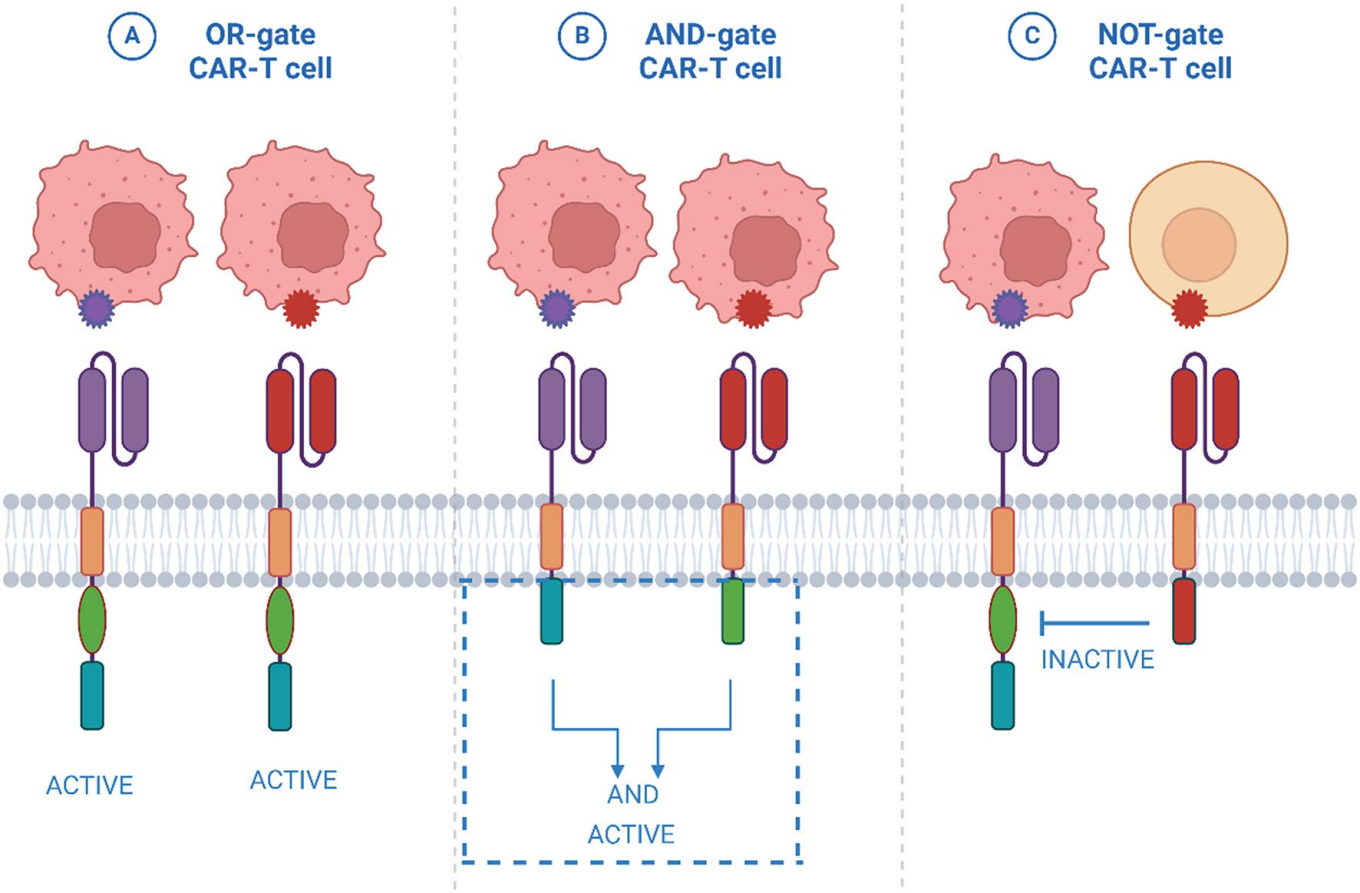
This figure sketches the structure of Boolean logic gates: (**A**) Shows the OR-logic gate that was developed to target more than one antigen in TME to overcome the tumor heterogenicity and antigen escape. (**B**) Shows the AND-Boolean logic gate that relies on the construction of two CARs targeting two different antigens and distributing the CD3 chain and the co-stimulatory molecules between the two receptors to link the activation of CAR-T cells to the presence of both antigens on the target cell. (**C**) Shows that The AND-NOT logic is another strategy to enhance tumor selectivity via incorporating of two different CARs on the T-cell; the first one is an iCAR that target a specific antigen on healthy cells and linked with an inhibitory signaling domain and the second one is activating CAR targeted against specific marker

Despite the promising efficiency of CAR T cell therapy in the treatment of cancer, it is linked to several toxicities such as TLS, GVHD, and off-tumor toxicity. So, it is obligatory to develop approaches to restrain the activity of CAR-T cells [51]. It’s common knowledge that the activity of CAR T-cells is affected by several variables including, the overall tumor mass, the antigen density on cancer cells, the affinity of the extracellular domain toward its target antigen, and finally the types of the costimulatory molecules incorporated in the CAR structure [52]. Consequently, modifying the CAR structure is one of the main tactics established to restrict the CAR T-cell activity. For instance, changing the amino acid sequence of the CD8 molecule in the hinge and transmembrane regions was shown to be associated with a lower cytokine release and CAR T-cell activity. As mentioned, the types of co-stimulatory molecules also play a role in regulating CAR T-cell activity. For example, CD28 co-stimulatory domain is characterized by a higher and rapid onset of expansion than 4-1BB which contributes to increase the risk of CAR T-cell toxicities. Based on that, 4-1BB is more suitable for patients with high tumor mass and antigen expression [53]. Boolean logic gates are considered one of the primary approaches utilized for boosting the safety and activity of CAR T cell therapy (Fig. 5). The first one is the AND-Boolean logic gate which is correlated with increasing the specificity of CAR T-cell therapy and limiting the off-tumor toxicity, as it requires the presence of two or more antigens to stimulate CAR T cells. The structure of this type of CAR T-cells relies on the construction of two CARs targeting two different markers and distributing the CD3 chain and the co-stimulatory molecules between the two receptors to link the activation of CAR-T cells to the presence of both antigens on the target cell so that the toxicity on healthy cells will be restricted [54, 55]. In a study conducted on pancreatic cancer models, they developed trivalent CAR T-cells that targets prostate stem cell antigen (PSA), IL-2, and transforming growth factor-B to improve CAR-T cell migration towards the cancer site and overcome the immunosuppressive environment [56]. The NOT logic is another strategy that was established to enhance tumor selectivity via incorporating two different CARs on the T-cell; the first receptor is an inhibitory CAR that targets a specific antigen on healthy cells and is linked with an inhibitory signaling domain and the second one is an activating CAR targeted against “tumor-associated antigen” on tumor cells [54].

The suicide gene is notably the most widely used strategy to limit CAR T-cell-associated toxicities. Herpes simplex virus thymidine kinase gene was reported to be inserted inside CAR T cells using viral or non-viral vectors to activate the prodrug ganciclovir and convert it to the active form via phosphorylation then, the active form will be incorporated within the DNA of the CAR T-cell and inhibit DNA synthesis. However, this way has some limitations including the inability to treat cytomegalovirus infection after stem cell transplantation and the long time taken to stimulate CAR T cell apoptosis. The expression of the caspase-9 gene with CAR is another novel approach to incite CAR T-cell apoptosis after the administration of a dimerizing agent such as AP1903, but this type of suicide gene causes permanent elimination of CAR-T cells [51, 57].

The Small molecule-assisted shut-off CAR (SMAsh CAR) is another approach designed to control the CAR localization on the T cell surface. This system is based on linking the c-terminal of the CAR protein with a protease-degron-based domain. This domain also contains a protease target site so, on the active state, the protease enzyme cleaves this target site resulting in separating the degradation moiety from the CAR structure. On the other hand, in order to induce CAR degradation, the patient administrates a protease inhibitor to hinder the protease enzyme activity and enable the degron moiety to destroy the CAR structure [52]. In a study conducted by Alexandre Juillerat et al., they embedded a protease-degron moiety within a second-generation CAR targeting CD22 and they found that upon the administration of a protease inhibitor called asunaprevir, the expression of the CAR protein on T cells and their killing efficacy were inhibited [58].

Dasatinib is a potent tyrosine kinase inhibitor that was established to be utilized in the treatment of Philadelphia chromosome-positive chronic myeloid leukemia due to its ability to inhibit BCR-ABL tyrosine kinase. It was demonstrated that dasatinib could be used as a safety switch to suppress the activity of CAR-T cells in a reversible manner because it is able to inhibit T-cell signaling via inhibiting lymphocyte-specific protein tyrosine kinase which phosphorylates CD3 chain; the intracellular domain in all CAR T cell generations, consequently leading to reversibly hamper T-cell proliferation and cytokine release [54, 59]. Katrin Mestermann et al. have reported that dasatinib is superior to glucocorticoids because it exerted complete and rapid onset in controlling CD19 CAR T-cell toxicities like; CRS and CAR-T cell-associated neurotoxicity, also the same results have been reported by Gabrielle Leclercq et al. [60, 61]. On the other hand, they assumed that dasatinib has some limitations; the first one is its inability to specifically target CAR T-cells, on the contrary, dasatinib represses the function of endogenous T cells too. The second limitation of dasatinib is related to its restricted ability to regulate the activity of CAR-T cells that have already been stimulated [59, 60].

### Combating Antigen Escape and Heterogeneity

Regarding hematological tumors, antigen escape is considered the main root cause of cancer resistance and relapse. Based on a study conducted using CAR T-cells targeting CD-19, it was demonstrated that the loss of antigen expression considered the leading cause of tumor resistance in 30–70% of r/r ALL patients. In contrast, solid tumors are specified by the presence of a variety of antigens expressed on cancer cells, thus rendering it challenging to eradicate tumors via targeting a single antigen. Currently, new technologies are being developed to target various antigens. Tandem CAR T-cell is one of these technologies generated via constructing the extracellular domain with two or more scFvs targeting two or more distinct antigens simultaneously [62]. A phase-1 clinical trial executed on LBCL patients using bispecific CAR T cells indicated that CD19/CD22 CAR T-cells exhibited favorable results in B-cell acute lymphoblastic leukemia patients with 82% MRD-CR and in LBCL patients with 62% ORR [63].

Going to more advanced approaches, universal CARs (UniCARs) were considered a novel strategy developed via transducing T-cells with the traditional CAR construct but have the capacity to recognize various antigens via binding to different linkers. These linkers are composed of labeled antibodies that act as a bridge between the intracellular signaling domains of CAR T-cells and target antigens on cancer cells [52, 64, 65]. For instance, the biotin-binding immune receptor (BBIR) is created by tagging the CAR construct with avidin which is able to bind specifically to the biotinylated antibodies that are designed to target several antigens. In a study carried out on trastuzumab-resistant tumor models, Lorinc Nagy et al. labeled CAR-T cells with monomeric streptavidin molecules (mSA2) to identify HER-2 via binding to the biotin-linked trastuzumab. Based on in vitro tests, they reported that UniCAR T cells could induce cell death in the core of tumor models via IFN-γ secretion similar to the conventional HER-2 CAR-T cells. However, according to the in vivo studies, xenograft mice started to die one week after UniCAR T-cell administration; this could be attributed to the on target off tumor toxicity caused by recognizing HER-2 in the lung tissues [66]. Based on a similar approach, the anti-fluorescein isothiocyanate CAR T-cells (Anti-FITC CAR T-cells) were developed to target different antigens by binding to FITC-linked antibodies [67].

### Disrupting the Immunosuppressive TME

Several clinical studies reported that CAR T-cell treatment accomplished notable success in blood malignancies unlike solid cancers due to the immunosuppressive environment which enhances CAR-T cell exhaustion via hypoxia-induced free radical production, continual antigen stimulation, and cholesterol-induced expression of programmed cell death receptors PD-1 [68]. There are several strategies to boost CAR T cell persistence by using gene editing techniques like CRISPR/Cas9 to delete genes responsible for T-cell exhaustion, several studies reported that the deletion of poly-ADP ribose polymerase-11 (PARP11) enhanced the expression of type-2 interferon receptor and cytotoxic activity of CAR T-cells [68].

PD-ligand overexpression is one of the main inhibitory mechanisms of cancer cells in TME. This ligand functions by recognizing the PD-1 receptor on infiltrating lymphocytes and hinders their killing efficacy against tumor cells. As a consequence, silencing of PD-1 gene is one of the primary approaches which may be applied by using CRISPR/Cas9 to enhance CAR T-cell cytotoxicity against malignancies [69]. Alternatively, instead of knocking out PD-1 through genetic alteration, R. S. Kalinin et al. utilized VHH antibody to prevent PD-1 localization on CAR T-cell surface and retain them within T-cells. They concluded that blocking the PD-1 expression could augment CAR T cell cytolytic activity, although it may diminish T-cell survival [70]. Rather than inhibiting PD-1 expression, replacing the inhibitory signaling domain of PD-1 with CD-28 co-stimulatory domain is another tactic that was applied by Hui Liu and his colleagues to enhance CAR T-cell killing efficiency. They reported that of 17 B cell lymphoma patients, 10 patients exhibited an objective response within three months and seven of them showed CR [71].

It is well known that cytokine secretion plays an important role in regulating immune response against cancer. So, one of the main strategies to counteract the immunosuppressive TME is to design the CAR construct with an additional capacity to express and secrete certain cytokines, this type of living product is known as TRUCK CAR T cells. IL-12, IL-18, and IL-15 are the most widely used cytokines that enhance the cytotoxic efficacy of TRUCK CAR-T cells and reduce tumor mass [72]. Additionally, TGF-β is another ligand that is secreted by cancer cells and accountable for inhibiting T-cell proliferation and inducing Treg to differentiate and secrete more TGF-β [72]. In a phase-1 clinical trial, CAR T-cells are transduced with a dominant negative TGF-β receptor-2 in addition to a CAR structure that targets (PSA). They revealed that of 13 metastatic prostate cancer patients, four patients exhibited ≥ 30% decrease in PSA expression with one of them achieving > 98% decreased PSA expression, unfortunately, five patients presented with grade ≥ 2 CRS [73]. Furthermore, CAR T cells may be developed to secrete bispecific T-cell engagers (BiTEs) which consist of two scFvs coupled with a linker. One of these two scFvs is designed to identify the CD3 molecule of T cells and the other is to target specific antigens on tumor cells so, they function as a bridge between T cells and cancer cells [74]. Blinatumomab is a CD3/CD19 BiTE and it was approved by the FDA to be efficient in treating B-cell malignancies. So, it may be promising to develop CAR T cell-secreting BiTEs to enhance their anti-tumor activity against cancer [72, 74]. Metabolic state reprogramming of T-cells during expansion is another approach that is used to elevate CAR-T cell activity. It was determined that the inhibition of pyruvate and glutamine uptake decreases T-cell exhaustion and promotes their differentiation into memory T-cells [68]. Conversely, raising potassium levels in TME and the pre-exposure of CAR-T cells to arginine may enhance their anticancer activity [52].

### CAR-N

Instead of using T cells to carry CARs, NK cells were reported to be a promising and safer candidate than T-cells because they have the ability to kill tumor cells with no need for MHC protein. for this reason, they are infrequent to induce GVHD [68, 75]. In addition to that, NK cells release a limited number of cytokines including IL-3 and granulocyte monocyte colony-stimulating factor, so it’s rare to induce CRS. This type of cell has another feature which is the ability to overcome the immunosuppressive TME due to their lower expression of PD-1 receptors [75]. Several studies revealed that CAR-NK cells are shown to impede tumor growth and increase survival rate in mouse models of BCL, HCC, breast, and prostate cancers [75–78].

## Conclusion

CAR-T cell therapy shows a propitious means of treatment for hematological as well as solid tumors pertaining to its shorter therapy duration and less severe side effects compared to its chemotherapy and radiotherapy alternatives. However, its efficiency in solid tumors is obstructed by the inadequacy of accurate antigen specificity by the CAR constructs on T cells, which causes tumor cells to escape from recognition, or worse, target healthy cells. Additionally, another set of limiting factors come to light upon targeting solid tumors, like the inability to properly traffick to, and infiltrate into the TME due to the continuous release of immunosuppressive cytokines. In the light of that context, several approaches were developed to augment CAR T cell specificity such as AND/NOT Boolean logic techniques, and to reduce CAR T cell toxicities via integrating a suicide gene in CAR-T cells or using a tyrosine kinase inhibitor. In the case of CAR T cell trafficking, expressing a CXCR2 receptor on CAR-T cells may enhance their infiltration to TME. Adding to the previous limitations, immunosuppressive TME could be resolved by designing CAR-T cells to produce specific cytokines, such as IL-12, to enrich the immune response against cancer. In conclusion, CAR-T cell therapy holds the capacity to be the long-awaited headline “Cancer Treatment” soon, but further research into how to enhance its efficacy and overcome its limitations is needed.

## Data Availability

Not applicable.

## Abbreviations

CSCs: Cancer stem cells
CAR T-cell: Chimeric antigen receptor T-cells
CD19 + EBV-LCL: Epstein-Barr virus-immortalized lymphoblastoid cell lines
SCFV: Single-chain fragment variable
MHC: Major histocompatibility complex
IL-12: Interlukine-12
MACS: Magnetic-Activated Cell Sorting
FACS: Fluorescence-Activated Cell Sorting
IFN: Interferon
Treg: T-regulatory cells
HCC: Hepatocellular carcinoma
EpCAM: Epithelial cell adhesion molecule
CLL-1: C-type lectin-like molecule-1
AML: Acute myeloid leukemia
LSCs: Leukemic stem cells
HSCs: Hematopoietic stem cells
CR: Complete response
MRD: Minimal residual disease negativity
GVHD: Graft versus host disease
TIM-3: T-cell immunoglobulin and mucin-domain containing-3
NF-KB: Nuclear factor-kappa beta
Siglec-6: Sialic acid-binding immunoglobulin-like lectin-6
FDA: Food and Drug Administration
EMA: European medicines agency
r/r NHL: Refracted/relapsed non-Hodgkin lymphoma
LBCL: Large B-cell lymphoma
Axi-cel: Axicabtagene ciloleucel
ORR: Objective responsive rate
SMAsh CAR: The Small molecule-assisted shut-off CAR
UniCARs: Universal CARs
BBIR: The biotin-binding immune receptor
HER-2: Human epidermal growth factor 2
BiTEs: Bispecific T cell engagers
Anti-FITC CARs: Anti-fluorescein isothiocyanate CAR
PSA: Prostate stem cell antigen
CRS: Cytokine release syndrome
Tisa-cel: Tisagenlecleucel
Liso-cel: Lisocabtagene maraleucel
BCMA: B cell maturation antigen
IL3Ra2: Interleukin-3 receptor subunit alpha-2
TME: Tumor microenvironment
CXCR2: C-X-C Chemokine receptor-2
CXCL-1: C-X-C Chemokine ligand-1
TGF + B: Transforming growth factor beta
iCAR: Inhibitory Chimeric antigen receptor T-cells
ALL: Acute lymphocytic leukemia
PD-1: Programmed cell death protein-1
CTLA-4: Cytotoxic T-lymphocytes associated protein-4
BCL: B-cell lymphoma
OS: Overall response
PFS: Progression-free survival
MUC-1: Mucin-1
C-Met: Mesenchymal-epithelial transition factor
PR: Partial response
CRi: Complete remission

## References

[1] Guo, Y., Feng, K., Wang, Y., & Han, W. (2018, Jun). Targeting cancer stem cells by using chimeric antigen receptor-modified T cells: a potential and curable approach for cancer treatment, 9*Protein Cell*(6), 516–526. 10.1007/S13238-017-0394-6 10.1007/s13238-017-0394-6 PMC5966354 28290053

[2] Yu, Z., Pestell, T. G., Lisanti, M. P., & Pestell, R. G. (2012, Dec.). Cancer stem cells. *International Journal of Biochemistry & Cell Biology*, *44*(12), 2144–2151. 10.1016/J.BIOCEL.2012.08.022 10.1016/j.biocel.2012.08.022 PMC3496019 22981632

[3] Cui, X., Liu, R., Duan, L., Cao, D., Zhang, Q., & Zhang, A. (2021, Nov.). CAR-T therapy: Prospects in targeting cancer stem cells, *J Cell Mol Med, 25*(21), 9891–9904. 10.1111/JCMM.16939 10.1111/jcmm.16939 PMC8572776 34585512

[4] Chen, L. S., Wang, A. X., Dong, B., Pu, K. F., Yuan, L. H., & Zhu, Y. M. (2012). A new prospect in cancer therapy: Targeting cancer stem cells to eradicate cancer. *Chin J Cancer*, *31*(12), 564. 10.5732/CJC.011.10444 22507219 10.5732/cjc.011.10444 PMC3777459

[5] Tan, S., Li, D., & Zhu, X. (2020, Apr.). Cancer immunotherapy: Pros, cons and beyond. *Biomedicine & Pharmacotherapy*, *124*, 109821. 10.1016/J.BIOPHA.2020.109821 10.1016/j.biopha.2020.109821 31962285

[6] Gauthier, J., & Yakoub-Agha, I. (2017, Sep. 01). Chimeric antigen-receptor T-cell therapy for hematological malignancies and solid tumors: Clinical data to date, current limitations and perspectives, *Current Research in Translational Medicine, 65*(3), (pp. 93–102). Elsevier Masson SAS. 10.1016/j.retram.2017.08.003 10.1016/j.retram.2017.08.003 28988742

[7] Ulmer, A. J., Scholz, W., Ernst, M., Brandt, E., & Flad, H. D. (1984). Isolation and subfractionation of human peripheral blood mononuclear cells (PBMC) by density gradient centrifugation on Percoll. *Immunobiology*, *166*(3), 238–250. 10.1016/S0171-2985(84)80042-X 6329947 10.1016/S0171-2985(84)80042-X

[8] Weiss, R., et al. (2021, Nov.). Comparison of three CD3-specific separation methods leading to labeled and label-free T cells. *Cells*, *10*(11). 10.3390/CELLS10112824/S1 10.3390/cells10112824 PMC8616525 34831046

[9] Milward, K., Hester, J., & Wood, K. J. (2019). Isolation of Human Regulatory T Lymphocytes by fluorescence-activated cell sorting. *Methods in Molecular Biology*, *1899*, 43–54. 10.1007/978-1-4939-8938-6_4 30649764 10.1007/978-1-4939-8938-6_4

[10] Ayala Ceja, M., Khericha, M., Harris, C. M., Puig-Saus, C., & Chen, Y. Y. (2024). CAR-T cell manufacturing: Major process parameters and next-generation strategies, 10.1084/jem.20230903 10.1084/jem.20230903 PMC10791545 38226974

[11] Zhang, C., Liu, J., Zhong, J. F., & Zhang, X. (2017, Jun.). Engineering, C. A. R. T. cells, *Biomark Res, 5*(1):1–6. 10.1186/S40364-017-0102-Y/FIGURES/2

[12] Wei, W., Chen, Z. N., & Wang, K. (2023, Aug.). CRISPR/Cas9: A Powerful Strategy to Improve CAR-T Cell Persistence, *Int J Mol Sci, 24*(15). 10.3390/IJMS241512317 10.3390/ijms241512317 PMC10418799 37569693

[13] Magnani, C. F., et al. (2020, Oct.). Sleeping beauty–engineered CAR T cells achieve antileukemic activity without severe toxicities. *J Clin Invest*, *130*(11), 6021. 10.1172/JCI138473 10.1172/JCI138473 PMC7598053 32780725

[14] Jayasooriya, V., Ringwelski, B., Dorsam, G., & Nawarathna, D. (2021, Oct.). mRNA-based CAR T-cells manufactured by miniaturized two-step electroporation produce selective cytotoxicity toward target cancer cells, *Lab Chip, 21*(19):3748–3761. 10.1039/D1LC00219H 10.1039/d1lc00219h PMC8513750 34585697

[15] Sudarsanam, H., Buhmann, R., & Henschler, R. (2022, Jun.). Influence of Culture conditions on Ex vivo expansion of T lymphocytes and their function for therapy: Current insights and open questions. *Frontiers in Bioengineering and Biotechnology*, *10*. 10.3389/FBIOE.2022.886637 10.3389/fbioe.2022.886637 PMC9277485 35845425

[16] Fda, & Cber (2017, October 18). Summary Basis for Regulatory Action - YESCARTA.

[17] KYMRIAH | FDA. Accessed: (2024, Jun. 01). [Online]. Available: https://www.fda.gov/vaccines-blood-biologics/cellular-gene-therapy-products/kymriah

[18] Śledź, M., Wojciechowska, A., Zagożdżon, R., & Kaleta, B. (2023, Dec.). In situ programming of CAR-T cells: A pressing need in Modern Immunotherapy. *Archivum Immunolgiae Et Therapiae Experimentalis*, *71*(1), 18. 10.1007/S00005-023-00683-Y 10.1007/s00005-023-00683-y PMC10329070 37419996

[19] Tokarew, N., Ogonek, J., Endres, S., von Bergwelt-Baildon, M., & Kobold, S. (2018, Nov.). Teaching an old dog new tricks: next-generation CAR T cells, *British Journal of Cancer 2018 120:1, 120*(1):26–37. 10.1038/s41416-018-0325-1 10.1038/s41416-018-0325-1 PMC6325111 30413825

[20] Qu, J., Mei, Q., Chen, L., & Zhou, J. (2021, Mar.). Chimeric antigen receptor (CAR)-T-cell therapy in non-small-cell lung cancer (NSCLC): Current status and future perspectives. *Cancer Immunology Immunotherapy*, *70*(3), 619–631. 10.1007/S00262-020-02735-0/TABLES/1 10.1007/s00262-020-02735-0 PMC7907037 33025047

[21] Yang, Y. H., Liu, J. W., Lu, C., & Wei, J. F. (2022). CAR-T cell therapy for breast Cancer: From Basic Research to Clinical Application. *International Journal of Biological Sciences*, *18*(6), 2609–2626. 10.7150/IJBS.70120 35414783 10.7150/ijbs.70120 PMC8990477

[22] Alhabbab, R. Y. (2020, Apr.). Targeting Cancer Stem cells by genetically Engineered Chimeric Antigen Receptor T Cells. *Frontiers in Genetics*, *11*, 517394. 10.3389/FGENE.2020.00312/BIBTEX 10.3389/fgene.2020.00312 PMC7188929 32391048

[23] Masoumi, J. (2021, Jul. 01). Cancer stem cell-targeted chimeric antigen receptor (CAR)-T cell therapy: Challenges and prospects, *Acta Pharmaceutica Sinica B, 11*(7):1721–1739. Chinese Academy of Medical Sciences. 10.1016/j.apsb.2020.12.015 10.1016/j.apsb.2020.12.015 PMC8343118 34386318

[24] Han, Y., Sun, B., Cai, H., & Xuan, Y. (2021). Simultaneously target of normal and stem cells-like gastric cancer cells via cisplatin and anti-CD133 CAR-T combination therapy, *Cancer Immunology, Immunotherapy, 70*(10):2795–2803, Oct. 10.1007/s00262-021-02891-x 10.1007/s00262-021-02891-x PMC10991976 33635343

[25] Zhang, B. L. (2019, Apr.). Preclinical Evaluation of Chimeric Antigen Receptor-Modified T Cells Specific to Epithelial Cell Adhesion Molecule for Treating Colorectal Cancer, *Hum Gene Ther, 30*(4), 402–412, 10.1089/hum.2018.229 10.1089/hum.2018.229 30693795

[26] Deng, Z., Wu, Y., Ma, W., Zhang, S., & Zhang, Y. Q. (2015, Jan.). Adoptive T-cell therapy of prostate cancer targeting the cancer stem cell antigen EpCAM. *Bmc Immunology*, *16*(1). 10.1186/s12865-014-0064-x 10.1186/s12865-014-0064-x PMC4318439 25636521

[27] Porcellini, S., et al. (2020, Feb.). CAR T cells redirected to CD44v6 Control Tumor Growth in Lung and Ovary Adenocarcinoma Bearing mice. *Frontiers in Immunology*, *11*. 10.3389/fimmu.2020.00099 10.3389/fimmu.2020.00099 PMC7010926 32117253

[28] Wang, H., et al. (2020). Minicircle DNA-mediated car T cells targeting cd44 suppressed hepatocellular carcinoma both in vitro and in vivo. *Onco Targets Ther*, *13*, 3703–3716. 10.2147/OTT.S247836 32440140 10.2147/OTT.S247836 PMC7210041

[29] Wang, J., et al. (2018, Jan.). CAR-T cells targeting CLL-1 as an approach to treat acute myeloid leukemia. *Journal of Hematology & Oncology*, *11*(1). 10.1186/s13045-017-0553-5 10.1186/s13045-017-0553-5 PMC5761206 29316944

[30] Zhang, H., Wang, P., Li, Z., He, Y., Gan, W., & Jiang, H. (2021, Jul.). Anti-CLL1 chimeric antigen receptor T-cell therapy in children with relapsed/refractory acute myeloid leukemia, *Clinical Cancer Research, 27*(13), 3549–3555, 10.1158/1078-0432.CCR-20-4543 10.1158/1078-0432.CCR-20-4543 33832948

[31] Lee, W. H. S. (2021, Sep.). Effective killing of acute myeloid leukemia by TIM-3 targeted chimeric antigen receptor T cells, *Mol Cancer Ther, 20*(9), 1702–1712, 10.1158/1535-7163.MCT-20-0155 10.1158/1535-7163.MCT-20-0155 34158344

[32] He, X. (2020). Bispecific and split CAR T cells targeting CD13 and TIM3 eradicate acute myeloid leukemia. [Online]. Available: https://ashpublications.org/blood/article-pdf/135/10/713/1717921/bloodbld2019002779.pdf 10.1182/blood.2019002779 PMC7059518 31951650

[33] Ferrari, B., & Peyvandi, F. (2020, Nov.). How I treat thrombotic thrombocytopenic purpura in pregnancy, *Blood, 136*(19), 2125–2132. 10.1182/BLOOD.2019000962 10.1182/blood.2019000962 32797178

[34] Neelapu, S. S. (2017, Dec.). Axicabtagene Ciloleucel CAR T-Cell Therapy in Refractory Large B-Cell Lymphoma, *New England Journal of Medicine, 377*(26), 2531–2544. 10.1056/nejmoa1707447 10.1056/NEJMoa1707447 PMC5882485 29226797

[35] Chen, Y. J., Abila, B., & Mostafa Kamel, Y. (2023, Feb. 01). CAR-T: What Is Next? *Cancers, 15*(3). MDPI. 10.3390/cancers15030663 10.3390/cancers15030663 PMC9913679 36765623

[36] Boardman, A. P., & Salles, G. (2023, Jun. 01). CAR T-cell therapy in large B cell lymphoma, *Hematological Oncology, 41*(S1), (pp. 112–118). John Wiley and Sons Ltd. 10.1002/hon.3153 10.1002/hon.3153 PMC10348487 37294963

[37] Schuster, S. J. (2019, Jan.). Tisagenlecleucel in Adult Relapsed or Refractory Diffuse Large B-Cell Lymphoma, *New England Journal of Medicine, 380*(1), 45–56, 10.1056/nejmoa1804980 10.1056/NEJMoa1804980 30501490

[38] Sterner, R. C., & Sterner, R. M. (2021, Apr.). CAR-T cell therapy: Current limitations and potential strategies. *Blood Cancer J*, *11*(4). 10.1038/S41408-021-00459-7 10.1038/s41408-021-00459-7 PMC8024391 33824268

[39] Majzner, R. G., & Mackall, C. L. (2018, Oct.). Tumor Antigen escape from CAR T-cell therapy. *Cancer Discovery*, *8*(10), 1219–1226. 10.1158/2159-8290.CD-18-0442 10.1158/2159-8290.CD-18-0442 30135176

[40] Salas-Benito, D., Berger, T. R., & Maus, M. V. (2023, Apr.). Stalled CARs: Mechanisms of Resistance to CAR T Cell Therapies, *7*, pp. 23–42. 10.1146/ANNUREV-CANCERBIO-061421-012235

[41] Sermer, D., & Brentjens, R. (2019, Jun.). CAR T-cell therapy: Full speed ahead, *Hematol Oncol, 37*(S1), (pp. 95–100). 10.1002/HON.2591 10.1002/hon.2591 31187533

[42] Jogalekar, M. P., Rajendran, R. L., Khan, F., Dmello, C., Gangadaran, P., & Ahn, B. C. (2022, Jul.). CAR T-Cell-Based gene therapy for cancers: new perspectives, challenges, and clinical developments, *Front Immunol, 13*. 10.3389/FIMMU.2022.925985 10.3389/fimmu.2022.925985 PMC9355792 35936003

[43] Luo, Y., Song, G., Liang, S., Li, F., & Liu, K. (2021, Mar.). Research advances in chimeric antigen receptor-modified T-cell therapy (review). *Exp Ther Med*, *21*(5). 10.3892/etm.2021.9915 10.3892/etm.2021.9915 PMC8005741 33790993

[44] Lin, H., Cheng, J., Mu, W., Zhou, J., & Zhu, L. (2021, Oct.). Advances in Universal CAR-T cell therapy. *Frontiers in Immunology*, *12*. 10.3389/fimmu.2021.744823 10.3389/fimmu.2021.744823 PMC8526896 34691052

[45] Ma, S., et al. (2019). Current progress in CAR-T cell therapy for solid tumors. *International Journal of Biological Sciences*, *15*(12), 2548–2560. 10.7150/IJBS.34213 31754328 10.7150/ijbs.34213 PMC6854376

[46] Li, J., Li, W., Huang, K., Zhang, Y., Kupfer, G., & Zhao, Q. (2018, Feb.). Chimeric antigen receptor T cell (CAR-T) immunotherapy for solid tumors: Lessons learned and strategies for moving forward. *Journal of Hematology & Oncology*, *11*(1). 10.1186/s13045-018-0568-6 10.1186/s13045-018-0568-6 PMC5809840 29433552

[47] Fedorov, V. D., Themeli, M., & Sadelain, M. (2013, Dec.). PD-1- and CTLA-4-based inhibitory chimeric antigen receptors (iCARs) divert off-target immunotherapy responses, *Sci Transl Med, 5*(215). 10.1126/SCITRANSLMED.3006597 10.1126/scitranslmed.3006597 PMC4238416 24337479

[48] Kosti, P., Maher, J., & Arnold, J. N. (2018, May 22). Perspectives on chimeric antigen receptor T-cell immunotherapy for solid tumors, *Frontiers in Immunology, 9*(22). Frontiers Media S.A. 10.3389/fimmu.2018.01104 10.3389/fimmu.2018.01104 PMC5972325 29872437

[49] van der Stegen, S. J. C. (2013, Nov.). Preclinical in vivo modeling of cytokinPD-1- and CTLA-4-based inhibitory chimeric antigen receptors (iCARs) divert off-target immunotherapy responsese release syndrome induced by ErbB-retargeted human T cells: identifying a window of therapeutic opportunity? *J Immunol, 191*(9)4589–4598. 10.4049/JIMMUNOL.1301523 10.4049/jimmunol.1301523 24062490

[50] Schubert, M. L. (2020). Side-effect management of chimeric antigen receptor (CAR) T-cell therapy, 10.1016/j.annonc.2020.10.478 10.1016/j.annonc.2020.10.478 33098993

[51] Yu, S., Yi, M., Qin, S., & Wu, K. (2019, Aug. 20). Next generation chimeric antigen receptor T cells: Safety strategies to overcome toxicity, *Molecular Cancer, 18*(1). BioMed Central Ltd. 10.1186/s12943-019-1057-4 10.1186/s12943-019-1057-4 PMC6701025 31429760

[52] Rafiq, S., Hackett, C. S., & Brentjens, R. J. (2020, Mar. 01). Engineering strategies to overcome the current roadblocks in CAR T cell therapy, *Nature Reviews Clinical Oncology, 17*(3). Nature Research, (pp. 147–167), 10.1038/s41571-019-0297-y 10.1038/s41571-019-0297-y PMC7223338 31848460

[53] Lin, H., Cheng, J., Mu, W., Zhou, J., & Zhu, L. (2021). Advances in Universal CAR-T cell therapy. *Frontiers in Immunology*, *12*. 10.3389/fimmu.2021.744823. Frontiers Media S.A. 10.3389/fimmu.2021.744823 PMC8526896 34691052

[54] Hong, M., Clubb, J. D., & Chen, Y. Y. (2020, Oct. 12). Engineering CAR-T Cells for Next-Generation Cancer Therapy, *Cancer Cell, 38*(4), (pp. 473–488). Cell Press. 10.1016/j.ccell.2020.07.005 10.1016/j.ccell.2020.07.005 32735779

[55] Han, X., Wang, Y., Wei, J., & Han, W. (2019, Nov. 29). Multi-antigen-targeted chimeric antigen receptor T cells for cancer therapy, *Journal of Hematology and Oncology, 12*(1). BioMed Central Ltd. 10.1186/s13045-019-0813-7 10.1186/s13045-019-0813-7 PMC6884912 31783889

[56] Sukumaran, S. (2018, Aug.). Enhancing the potency and specificity of engineered T cells for cancer treatment, *Cancer Discov, 8*(8), (pp. 972–987). 10.1158/2159-8290.CD-17-1298 10.1158/2159-8290.CD-17-1298 PMC6428579 29880586

[57] Stock, S. (2023). Mechanisms and strategies for safe chimeric antigen receptor T-cell activity control, *International Journal of Cancer*. John Wiley and Sons Inc, 10.1002/ijc.34635 10.1002/ijc.34635 37350095

[58] Walcher, L. (2020, Aug. 07). Cancer Stem Cells—Origins and Biomarkers: Perspectives for Targeted Personalized Therapies, *Frontiers in Immunology, 11*. Frontiers Media S.A., 10.3389/fimmu.2020.01280 10.3389/fimmu.2020.01280 PMC7426526 32849491

[59] Zheng, Y., Nandakumar, K. S., & Cheng, K. (2021, Jul. 22). Optimization of CAR-T Cell-Based Therapies Using Small-Molecule-Based Safety Switches, *Journal of Medicinal Chemistry, 64*(14), (pp. 9577–9591). American Chemical Society. 10.1021/acs.jmedchem.0c02054 10.1021/acs.jmedchem.0c02054 34191515

[60] Mestermann, K. et al. (2019). The tyrosine kinase inhibitor dasatinib acts as a pharmacologic on/off switch for CAR T cells. [Online]. Available: https://www.science.org 10.1126/scitranslmed.aau5907 PMC7523030 31270272

[61] Leclercq, G., et al. (2021, Jul.). Src/lck inhibitor dasatinib reversibly switches off cytokine release and T cell cytotoxicity following stimulation with T cell bispecific antibodies. *Journal for Immunotherapy of Cancer*, *9*(7). 10.1136/jitc-2021-002582 10.1136/jitc-2021-002582 PMC8323395 34326166

[62] Sterner, R. C., & Sterner, R. M. (2021, Apr. 01). CAR-T cell therapy: current limitations and potential strategies, *Blood Cancer Journal, 11*(4). Springer Nature. 10.1038/s41408-021-00459-7 10.1038/s41408-021-00459-7 PMC8024391 33824268

[63] Spiegel, J. Y. (2021, Aug.). CAR T cells with dual targeting of CD19 and CD22 in adult patients with recurrent or refractory B cell malignancies: a phase 1 trial, *Nat Med, 27*(8), 1419–1431. 10.1038/s41591-021-01436-0 10.1038/s41591-021-01436-0 PMC8363505 34312556

[64] Zhao, J., Lin, Q., Song, Y., & Liu, D. (2018, Nov. 27). Universal CARs, universal T cells, and universal CAR T cells, *Journal of Hematology and Oncology, 11*(1). BioMed Central Ltd. 10.1186/s13045-018-0677-2 10.1186/s13045-018-0677-2 PMC6257951 30482221

[65] Minutolo, N. G., Hollander, E. E., & Powell, D. J. J. (2019). The emergence of universal immune receptor t cell therapy for cancer, *Frontiers in Oncology, 9*. Frontiers Media S.A. 10.3389/fonc.2019.00176 10.3389/fonc.2019.00176 PMC6448045 30984613

[66] Nagy, L., Mezősi-Csaplár, M., Rebenku, I., Vereb, G., & Szöőr, Á. (2024). Universal CAR T cells targeted to HER2 with a biotin-trastuzumab soluble linker penetrate spheroids and large tumor xenografts that are inherently resistant to trastuzumab mediated ADCC. *Frontiers in Immunology*, *15*. 10.3389/fimmu.2024.1365172 10.3389/fimmu.2024.1365172 PMC10982377 38562932

[67] Lee, Y. G., et al. (2019, Jan.). Use of a single CAR T cell and several bispecific adapters facilitates eradication of multiple antigenically different solid tumors. *Cancer Research*, *79*(2), 387–396. 10.1158/0008-5472.CAN-18-1834 10.1158/0008-5472.CAN-18-1834 30482775

[68] Dianat-Moghadam, H., Sharifi, M., Salehi, R., Keshavarz, M., Shahgolzari, M., & Amoozgar, Z. (2023, Feb. 01). Engaging stemness improves cancer immunotherapy, *Cancer Letters, 554*. Elsevier Ireland Ltd. 10.1016/j.canlet.2022.216007 10.1016/j.canlet.2022.216007 36396102

[69] McGowan, E., Lin, Q., Ma, G., Yin, H., Chen, S., & Lin, Y. (2020, Jan. 01). PD-1 disrupted CAR-T cells in the treatment of solid tumors: Promises and challenges, *Biomedicine and Pharmacotherapy, 121*. Elsevier Masson SAS, 10.1016/j.biopha.2019.109625 10.1016/j.biopha.2019.109625 31733578

[70] Kalinin, R. S., et al. (2021, Oct.). Engineered removal of PD-1 from the surface of CD19 CAR-T cells results in increased activation and diminished survival. *Front Mol Biosci*, *8*. 10.3389/fmolb.2021.745286 10.3389/fmolb.2021.745286 PMC8548718 34722633

[71] Liu, H., et al. (2021, Jan.). CD19-specific CAR T cells that express a PD-1/CD28 chimeric switch-receptor are effective in patients with PD-L1⇓positive B-cell lymphoma. *Clinical Cancer Research*, *27*(2), 473–484. 10.1158/1078-0432.CCR-20-1457 10.1158/1078-0432.CCR-20-1457 33028589

[72] Hawkins, E. R., D’souza, R. R., & Klampatsa, A. (2021). Armored CAR T-cells: The next chapter in T-cell cancer immunotherapy, *Biologics: Targets and Therapy, 15*. Dove Medical Press Ltd (pp. 95–105). 10.2147/BTT.S291768 10.2147/BTT.S291768 PMC8053711 33883875

[73] Narayan, V., et al. (2022, Apr.). PSMA-targeting TGFβ-insensitive armored CAR T cells in metastatic castration-resistant prostate cancer: A phase 1 trial. *Nature Medicine*, *28*(4), 724–734. 10.1038/s41591-022-01726-1 10.1038/s41591-022-01726-1 PMC10308799 35314843

[74] Zhou, S., Liu, M., Ren, F., Meng, X., & Yu, J. (2021, Dec. 01). The landscape of bispecific T cell engager in cancer treatment, *Biomarker Research, 9*(1). BioMed Central Ltd. 10.1186/s40364-021-00294-9 10.1186/s40364-021-00294-9 PMC8157659 34039409

[75] Wang, W., Jiang, J., & Wu, C. (2020, Mar. 01). CAR-NK for tumor immunotherapy: Clinical transformation and future prospects, *Cancer Letters, 472*, (pp. 175–180). Elsevier Ireland Ltd. 10.1016/j.canlet.2019.11.033 10.1016/j.canlet.2019.11.033 31790761

[76] Chen, X., et al. (2016). A combinational therapy of EGFR-CAR NK cells and oncolytic herpes simplex virus 1 for breast cancer brain metastases. *Oncotarget*, *7*(19). 10.18632/oncotarget.8526 10.18632/oncotarget.8526 PMC5053686 27050072

[77] Oelsner, S., et al. (2017). Continuously expanding CAR NK-92 cells display selective cytotoxicity against B-cell leukemia and lymphoma. *Cytotherapy*, *19*(2). 10.1016/j.jcyt.2016.10.009 10.1016/j.jcyt.2016.10.009 27887866

[78] Chang, Y. H., Connolly, J., Shimasaki, N., Mimura, K., Kono, K., & Campana, D. (2013). A chimeric receptor with NKG2D specificity enhances natural killer cell activation and killing of tumor cells. *Cancer Research*, *73*(6). 10.1158/0008-5472.CAN-12-3558 10.1158/0008-5472.CAN-12-3558 23302231

